# Gender Matters: Understanding Transitions in Surgical Education

**DOI:** 10.3389/fmed.2022.884452

**Published:** 2022-05-10

**Authors:** Gozie Offiah, Stuart Cable, Charlotte E. Rees, Susie J. Schofield

**Affiliations:** ^1^Royal College of Surgeons in Ireland, University of Medicine and Health Sciences, Dublin, Ireland; ^2^Centre for Medical Education, School of Medicine, University of Dundee, Dundee, United Kingdom; ^3^School of Health Sciences, College of Health, Medicine and Wellbeing, The University of Newcastle, Callaghan, NSW, Australia; ^4^Faculty of Medicine, Nursing and Health Sciences, Monash Centre for Scholarship in Health Education, Monash University, Clayton, VIC, Australia

**Keywords:** gender, transitions, surgical training, surgical education, surgical career

## Abstract

**Introduction:**

Diverse transitions are elemental to medical career trajectories. The effective navigation of such transitions influences a sense of belonging and wellbeing, positive relationships, and good engagement and attainment within new contexts. Using Multiple and Multidimensional Transitions (MMT) theory as an analytical lens, this paper aims to answer the research question: “What gendered transitions do female surgeons experience, and how do these gendered transitions impact them?”

**Methods:**

We conducted a qualitative study drawing on narrative inquiry, with face-to-face and online semi-structured interviews with 29 female surgeons across nine surgical specialities in Ireland and Scotland. This paper is part of a larger study including male surgeons, other colleagues and patients of female surgeons. The female surgeons in this paper were purposively sampled using maximum variation sampling across several levels (consultants, trainees and middle-grade doctors), as well as six who had transitioned out of surgery. Framework analysis was employed to interrogate the interview data.

**Results:**

Five overarching types of transitions were identified across surgical education but only three of these transitions—work, culture and health—were primarily experienced by female surgeons (not male surgeons so were considered gendered), thereby impacting social, academic, and psychological domains. The remaining two types of transition—education and geography—were seemingly experienced equally by female and male surgeons, so are beyond the scope of this paper focused on female surgeons’ gendered experiences.

**Conclusion:**

This novel qualitative study drawing on MMT theory illustrates how multiple gendered transitions interact and impact female surgeons across the surgical education continuum. Aligned with MMT theory, family members and others are also purportedly affected by female surgeons’ transitions. Healthcare educators, leaders and policymakers need to better understand gendered transitions and their impacts to improve support for female surgical trainees on their educational journeys.

## Introduction

Transitions in medical education are extensively cited in the literature, explained through different models and theories, including Multiple and Multidimensional Transitions (MMT) theory and several of Vygotsky’s theories (1–6). Of particular interest to medical educators are transitions from preclinical to clinical medical student (4, 7), from final year medical student to doctor (8–10), and from specialist trainee to consultant (11, 19). While the focus has been on transitions across different stages of training, very little has been published on surgical education transitions, considering the multiplicity and multidimensionality of transitions and their interrelations with female gender.

### Multiple and Multidimensional Transitions Theory

Jindal-Snape (1) first introduced MMT theory in educational contexts to better understand transitions. Looking at international students’ transitions, Jindal-Snape suggested that transitions were many and multidimensional, with the physical, human and social environment playing key roles in transition experiences. MMT theory highlights the multiple domains involved in transitions, including physical, cultural, psychological and social. Jindal-Snape highlights that individuals will experience transitions, for example, moving to a new organisation (affecting their physical domain) or transitions related to relationships with colleagues, patients, family and friends (affecting their psychological and social domains). Whilst one domain might remain constant, others can be in a state of flux, and these multiple domains create complex interrelationships. MMT theory also states that individuals’ transitions can trigger transitions for their significant others and vice-versa (12). Moreover, a change in one domain often triggers changes in other domains, e.g., a change in working relationships with colleagues in the social domain may trigger positive and negative changes in the psychological domain.

### Medical Transitions

Transitions are individual, social and contextual processes (13). Westerman and Teunissen [(14), p. 372] define a medical transition as “a period of change in which medical students or doctors experience a discontinuity in their professional life space forcing them to respond by developing new behaviours or changing their professional life space to cope with a new situation.” Only a few recent studies in healthcare have used MMT theory to better understand medical transitions. These include a Scottish study of the trainee-to-trained doctor transition, including the development of a conceptual model of the trainee-trained doctor transition (15, 16, 19), an Australian study exploring new healthcare graduates’ transitions (17) and an English study exploring new doctors’ transitions to practice utilising participant-voiced poetry (18). Most relevant to the current paper, Gordon et al. (16) developed the Transition-To-Trained Doctor (T3D) model to reflect the complexity of how doctors experience trainee-trained doctor transitions, taking into account various personal and professional domains and contexts. MMT theory argues that transitions are complex, iterative and affect other changes. A related study by Gordon et al. (5) identifies multiple intersecting transitions triggered by the pandemic in multiple contexts, challenging the notion of transitions as simple and linear. While Gordon et al. (16, 19) hinted at the importance of gendered transitions in their longitudinal case study of a female surgeon who was transitioning through surgical training (trainee doctor) to consultant (trained doctor), none of these studies thus far have specifically examined the impacts of the female gender on these transitions. There is a lack of literature on gendered transitions and hence the importance of further exploring female gender in this paper.

### Female Gender and Surgical Training

One of the most significant changes for women in surgery in the twenty-first century is increased numbers of women studying medicine, with women typically making up at least 50% of graduating medical school classes (20). However, the number of women pursuing surgical careers is still very low, with causative explanations including gender-based bullying, gender discrimination, harassment, and lack of mentoring and role models in surgical practice (21–25). In addition to these barriers, studies have identified transition challenges for female surgical trainees. Multiple types of transitions experienced by female surgeons include temporal (e.g., into higher education, clinical learning, clinical practice and clinical leadership) and spatial (e.g., urban-rural, clinician-academic) (26). There are also transitions at the individual, interpersonal and organisational levels. For example, a change in one’s health can trigger a non-normative life transition leading to changes in identity, status, interactions and relationships, beliefs and values, requiring substantial psychosocial and cultural adaptation (1, 12). Indeed, recognising that one type of transition is probably one of many experienced by trainees is important. Demonstrating this multidimensionality, research across the medical education continuum has shown that transitions impact the individual, interpersonal relationships, careers and the system, resulting in raised stress levels, negative emotions and burnout (27, 28). With these multiple and multidimensional elements of transitions, it has been shown that doctors cannot be fully prepared for the transitions into all aspects of their work (29).

## Study Aims and Research Questions

This study addresses research gaps identified in the literature on gendered transitions for female surgeons. It offers novel insights by exploring in-depth interviews with female surgeons as they reflect on *all* transitions, not one specific transition at a specific stage of training, as other research has tended to do (15, 16, 18, 19). Furthermore, the current paper is speciality-specific, focusing on surgical educational transitions, and it includes transitions across surgical speciality training to consultant level, as well as female surgeons transitioning out of surgery rather than only at the start or end of training. It is a multi-country study (Ireland and United Kingdom) and adopts a novel gender- and transitions-focused lens. Using MMT theory as an analytical lens, this paper aims to answer the research question: “What gendered transitions do female surgeons experience, and how do these gendered transitions impact them?”

## Materials and Methods

### Study Design

We used a qualitative study drawing on narrative inquiry, with face-to-face and online semi-structured interviews underpinned by social constructionism, which asserts that people construct meaning as they interact with their worlds (30). The study draws on MMT theory (1) as the analytical lens to explore female gendered transitions across the surgical training continuum.

### Context

This study was conducted within surgical specialities in Ireland and Scotland. Within Scotland in the United Kingdom, the surgical training programme involves completing a 2-year core surgical training, with trainees’ progress being monitored regularly, and completion of the programme depending upon satisfactory outcomes at the Annual Review of Competence Progression (ARCP). An interview follows this to enter the specialist training programme of 4–6 years, depending on the surgical subspecialty. In comparison, Ireland’s National Surgical Training Programme runs an 8-year training programme. The first two years (similar to the Scottish system) consist of six months of general surgery, 6 months within another speciality in the first year, and speciality-specific rotations in the second year. Trainees are assessed via the Competency Assessment and Performance Appraisal (CAPA) throughout the two years. The remaining six years of training is focused on surgical subspecialty training. At the end of the training, trainees are awarded a Certificate of Completion of Specialist Training in both countries, allowing them to become surgical consultants. In both Scotland and Ireland, training occurs within a public healthcare system, where care is free at the point of delivery.

### Sampling and Participants

Participants were purposively sampled employing maximum variation sampling from six hospital groups in Ireland and seven health boards in Scotland (31). We recruited participants through emails, posters, snowballing, and through a clinical reference group (see “Acknowledgments” section). Twenty-nine female surgeons from Ireland and Scotland participated in this study. The maximum variation sampling helped to identify diverse views and experiences (32). For example, female surgeons are more likely to work in certain surgical specialities like paediatrics, obstetrics and gynaecology, and plastics (33–36). In contrast, male-dominated specialities include orthopaedics, neurosurgery and thoracic surgery (37–41). Therefore, it was essential that recruitment included participants from various specialities to explore diversity across both female- and male-dominated surgical specialities. Table 1 illustrates the range included in this study across nine different surgical specialities in both countries, interviewees’ different grades and training stages. We received ethical approval from six sites in Ireland and one overarching approval in Scotland.

**TABLE 1:** Participant characteristics.

Characteristics	Female surgeons
**Country:**	
Ireland	7
Scotland	22
**Ethnicity:**	
White	23
Middle Eastern	2
Asian	4
**Age range:**	
30–39	15
40–49	11
50–59	3
**Speciality**	
Breast surgery	4
Colorectal surgery	2
General surgery	4
Orthopaedic surgery	4
Paediatric surgery	2
Plastic surgery	8
Transplant surgery	2
Upper gastrointestinal surgery	1
Vascular surgery	2
**Level of training**	
Consultants	8
Trainees	13
Staff grade*	2
Transition out of surgery	6

*Staff grade doctors have permanent positions as middle-grade doctors. They work under consultants and must have more than 6 years’ experience in a speciality (www.bma.org.uk).

*Male surgeons’ characteristics can be found in Offiah (42).*

*Participants provided their self-identified gender, and all participants in the larger study identified as either female or male.*

### Data Collection

Data collection occurred between November 2016 and April 2019, as part of a wider study involving 60 interviews with female and male surgeons (only female surgeons’ data are presented in this paper), female and male colleagues (e.g., anaesthetists, nurses and physician associates), and male and female patients. Table 1 provides the demographics of female surgeons only [as they are the focus of this paper; the demographic characteristics of all sixty participants can be found in Offiah (42)]. A semi-structured approach to interviewing was adopted with the same researcher (GO) conducting all interviews. GO used a narrative interview method to elicit participants’ experiences. Participants were initially asked to recount their surgical education journeys, from their undergraduate medical education to postgraduate training and, where relevant, to consultant surgeons. Table 2 provides an example of questions used. All interviews were audio-recorded with permission, and audio files were transcribed. Participants were assigned unique identifiers to maintain their anonymity.

**TABLE 2:** Questions used during interviews.

Questions used in the narrative interview
Please tell me the story of your life as a surgeon beginning with your medical training from medical school to postgraduate training and where relevant to surgeon consultant. Please include all events and experiences that have been important to you personally.
Please describe your own most memorable stories of these key experiences along your surgical path.

Participant numbers for our current study provided sufficient *information power* given that we collected 36h of rich, in-depth interview data (43). The study had a focused aim (i.e., to explore female stakeholders’ lived experiences of gender in surgery), tight sample specificity (i.e., female surgeons), use of established theories [i.e., MMT theory (1)], high-quality researcher-participant dialogue via in-depth interviewing and an in-depth, team-based approach to data analysis (43, 44). Appraisal of information power was conducted throughout the analysis process, beginning with SS and CER reviewing three initial interviews, with interviewing technique affirmation and feedback provided to author GO to further enhance interview dialogue.

### Data Analysis

The dataset was analysed using a five-step Framework Analysis approach (45), including familiarisation, framework development, indexing, charting and mapping. We (GO, SS, CER, and SC) participated in this inductive process, beginning with identifying key themes within a subset of data. Listening to audios and reading transcripts, GO coded the entire data using NVivo, with SS, SC, and CER checking portions of coding. The team discussed and agreed upon new themes and sub-themes identified through the analytic process. The final framework was written and defined in an 18-page coding document (available on request from the corresponding author), which comprised five themes based on *what* participants said and one theme based on *how* events were narrated. As mentioned above, the data were examined cross-sectionally through the lens of MMT theory (1).

### Qualitative Rigour

We established rigour through ensuring team reflexivity, internal coherence and crystallisation. A team reflexivity exercise enabled us to better understand research team members’ perspectives, thereby contributing to a more rigorous analysis process (46). The first author of this paper is a female surgeon of colour. Our analysis team of three other researchers included two further females and one male. Two of our four-person team have clinical backgrounds, and two are non-clinical health professions education experts. We range in our levels of experience with qualitative methodologies and gender research (one expert, two intermediates, one novice). Internal coherence, an essential element of research quality, refers to the alignment between ontology, epistemology, methodology and methods in qualitative research (47). We achieved this internal coherence by ensuring alignment between our relativist ontology, constructionist epistemology, interpretivist qualitative methodology, and narrative interview methods. Finally, crystallisation embraces multiple realities and its use ensures methodological rigour within qualitative approaches. For this study, crystallisation ensured data completeness by highlighting the authors’ positionality within the research, the bringing together of multiple theories, multiple researchers, and reflexivity (48).

## Results

**FIGURE 1:**
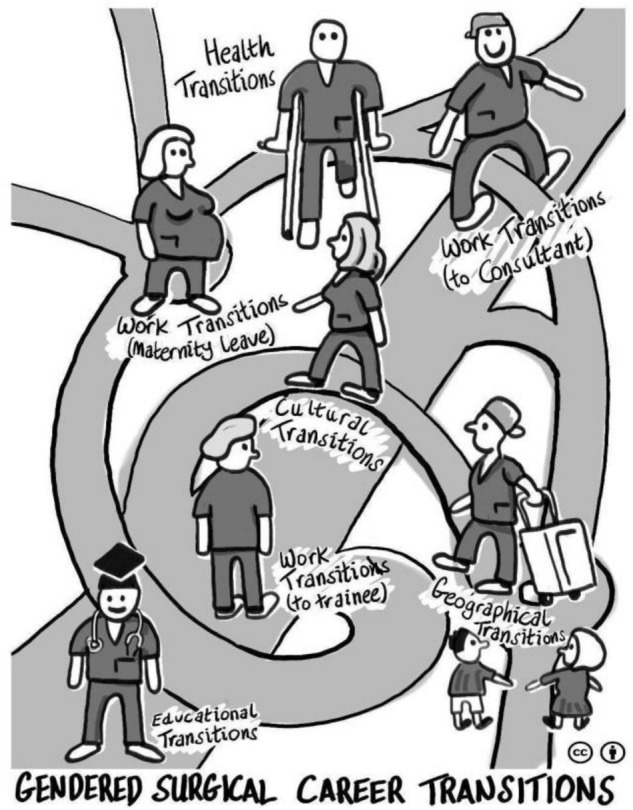
Illustration of multiple transitions.

Transitions were identified as individual, social and contextual processes in our Scottish and Irish data. The female surgeons described numerous transitions triggered by their surgical training, identifying these as the most challenging within their medical career trajectories, with gender having considerable impacts on their surgical training and careers. Five overarching themes related to transitions were identified from our framework analysis, three of which were highly gendered for females (work, culture and health), as they were only narrated by females and not male surgeons in our larger study. The two remaining transitions (education and geography) did not appear gendered for females as they were reported by male *and* female surgeons, and are therefore outside the scope of this paper focusing on gendered transitions for female surgeons [see (42) for an articulation of male surgeons’ experiences]. The three female gendered themes presented in this paper are interrelated (despite being presented separately) because one type of transition often triggers another type of transition, e.g., work transitions trigger cultural transitions; health transitions trigger work transitions, etc. See Figure 1 for a visual representation of all five transition themes.

## Gendered Work Transitions for Females

Gendered work transitions were the primary theme identified from our data relevant to this paper. Four key female gendered work transitions were identified: maternity leave transitions; transitions to part-time training/working; leadership transitions; and transitions out of surgery. Drawing on MMT theory highlighted how participants depicted transitions in multiple contexts, including workplace, home, education and their role as surgeons, as noted by illustrative quotes below.

### Maternity Leave Transitions

Returning to work post-maternity leave was challenging for many female surgeons, especially considering the requirement to maintain surgical competence despite being absent from the surgical workplace, sometimes for 6–12 months. Several female trainees expressed concerns about post-maternity transitions to work given colleagues’ high expectations of their performance. One female surgeon narrated her challenging transition from maternity leave into surgical practice and her professional life as a surgeon more broadly:

“I have to say yeah, coming back after maternity leave, I can remember holding the retractors thinking, “What am I doing here?” Really having very little to talk to with other people and feeling like an absolute fish out of water… You know, moving from childcare 24-h a day to… holding retractors [Surgical instruments used for exposure]. It’s a very difficult time, I think that transition, coming back in after being out for a year with family. I imagine that a lot of female doctors would be lost in that transition.” ID36, Scottish Female Staff Grade Surgeon, Aged 40–49.

This female surgeon narrates the complex nature of transitions between motherhood and professional life as a surgeon. Other female surgeons reported this new mother-surgeon transition as difficult, impacting other aspects of their lives (e.g., perceptions of their new identity as a mother) and their family’s lives (e.g., impacts on relationships with family or the additional need to cater for childcare to fit the family situation). Post-maternity leave, there was the additional transition of childcare, with very little support from the surgical system, demonstrating the sacrifices required throughout females’ surgical training and the negative impacts on female surgeons (e.g., missing their children growing up as they have to work long hours), their children (e.g., constant moving of schools or childcare to accommodate surgical training) and careers (e.g., surgical cultures valuing presenteeism could be problematised for a working mother).

### Part-Time Training/Working Transitions

Several female surgeons described transitions to part-time training (or working), explaining the challenges of no longer being full-time trainee (or surgeon). This transition often intersected with other transitions for surgical trainees (e.g., having babies) and their colleagues (e.g., increased workloads). In addition, working part-time meant that trainees were only present on certain weekdays, creating significant impacts around team-working, including rapport-building with colleagues and colleagues’ behaviours towards them, such as being excluded from team activities:

“In the beginning, even though we had a lot of hours, I still felt like I was a valued member of the team and that I was respected and that I felt part of it, and I think as I have gone on through my career, I have been feeling less like that, and that is an issue, and I don’t know whether that is being less than full time.” ID26, Scottish Female Surgical Trainee, Aged 30–39.

Female surgeons undertaking part-time training highlighted the multidimensional impacts involving the individual (e.g., impact on their self-confidence) and their families, their colleagues (e.g., relationships within the team) and the surgical system. While working part-time afforded trainees more time with their families, it negatively affected their own work-life, as well as their families, colleagues and the surgical system itself. For example, while some female trainees appreciated the positive nature of part-time work, the negative impacts of part-time training included disrespect from colleagues, lower salary, not being perceived as a full member of the team and extending the length of training.

### Leadership Transitions

Many participants discussed expected transitions to becoming surgical consultants. These transitions involved moving to senior roles, either within the same or new workplaces. Female surgeons identified dealing with new workplace transitions (e.g., new systems) on becoming consultants and with changing expectations. In addition, female surgeons narrated the challenges of decision-making in applying for consultant roles, given the impacts such a role would have on their personal and family lives:

“I’m coming to the point where applying for consultant posts or going onto another fellowship… where I would love to just be able to do a fellowship in (names local city)… But that means that my husband has a year of single parenting in front of him… after all these years of training, all this time of moving. He’s built up another business, and it’s just coming to fruition. So, he’s not at all impressed with that idea of me going somewhere else. I have to make a decision against my career.” ID54, Scottish Female Surgical Trainee, Aged 40–49.

This female trainee explicated her decision-making processes in her impending transition to consultant and potential negative impacts on her family life. Her need to consider her husband’s business and him potentially single parenting their children offers a stark example of the complex nature of leadership transitions, where female surgeons’ careers impacted significant others’ transitions.

### Transitions Out of Surgery

Six interviewees reported having resigned from surgery and thus narrated transitions to different specialities. Several of these female surgeons were near the end of their training. They reported challenges with work-life balance in their surgical training and a lack of support from senior surgeons as reasons for leaving surgery, indicating systemic causes of surgical attrition:

“I miss being in the theatre with the nurses at 2:00 a.m. doing an appendix. I didn’t miss being unsupported with complex operations… The hours never really bothered me. I work as many hours now as I did then. The lack of support is, I think, that was maybe… the final nail in the coffin.” ID80, Irish Resigned Female Surgeon, Aged 30–39.

This female ex-surgeon does not problematise expected presenteeism (e.g., long hours), common to surgery, but rather the lack of support, illustrating her transitions intertwining psychological and social dimensions. In this example, she employs strong emotional and metaphoric language (“*final nail in the coffin*”) to express the psychological factors triggering the death of her surgical career. Another female ex-surgeon narrated the challenges she faced as a surgical trainee, which contributed to her resigning from surgery. She reported the negative influences on her family life (she was pregnant, had another little child at home and was asked to move to the south of the country for training). She described being offered a surgical training position but declined it due to the multiple transitions required within the training programme and the potential negative impacts on her family life. Participants within this cohort often narrated their needs for supportive networks as they tried to manage the impact of these work transitions.

## Gendered Cultural Transitions for Female Surgeons

Several female surgeons reported transitioning to more western countries, impacting their personal and professional identities. Female surgeons who identified as International Medical Graduates (IMGs) described experiencing multiple cultural transitions, such as transitioning to westernised cultures of independence and away from more collectivist societies. Their talk emphasised the cultural and psychological changes resulting from contacts with different cultural groups and their members, some of which were positive:

“It didn’t matter [in Ireland] if you are dressed conservatively or dressed in a more liberal way, as long as you were doing the right thing and taking the right decisions, and that really impressed me, especially coming from a country where you are judged for simple things from your appearance to whether you speak too much English (laughs).” ID38, Irish Female Surgeon Staff Grade, Aged 40–49.

While this example is positive, other female IMGs narrated challenges adapting to new societies (e.g., the need to understand workplace norms to progress their careers) and the negative impacts on their personal (e.g., perceptions of racism in the work environment) and professional lives (e.g., the inability to secure a numbered training post). Adapting to cultural differences was a significant challenge for these female doctors as they transitioned from their countries. Other female participants noted their experiences of struggling to secure a surgical post as an overseas doctor because of the perceived discrimination within the system, for example, a white male candidate getting jobs ahead of a more experienced female surgeon of colour.

### Gendered Health Transitions for Females Surgeons

Female surgeons’ health triggered other transitions in their lives and careers, impacting their professional identities. Some female surgeons reported the challenges of watching peers progress while unwell and being discriminated against because of their health conditions, adversely affecting their surgical training transitions.

“I became unwell about 3 years before I was due to become a consultant. And then found myself unable to work full-time because I had complications after surgery. So, I worked 4 days a week, initially doing on-calls and then not doing on-calls. I then [had another health event], even worse at that point. Still stayed on doing 4 days a week but watched all my peers getting ahead of me. Things weren’t going very well with how people were treating me because I was called the ‘the part-time woman’ who refused to do on-calls, so that made life a bit difficult.” ID31, Scottish Resigned Female Surgeon, Aged 40–49.

Health transitions often necessitated work transitions like working part-time and not doing on-call rather than fulfilling the traditional expectation of full-time working and doing on-calls (presenteeism mentioned earlier). This powerful quote from a female ex-surgeon described how her health transitions adversely affected her surgical training transitions. She reported the challenges of being treated unfavourably compared with her peers. Interestingly, this quote clarifies how her health transition necessitated her work transitions, working part-time rather than full-time. As well as indicating her health stressors, this quote also illustrates associated sociocultural stressors, including discrimination. These transitions are therefore interrelated. As a result of her ill health, she worked four days a week, which led to peers getting ahead of her and negatively impacting her chances of becoming a consultant. Our findings demonstrated that ill health in female surgeons triggered life transitions, leading to changes in their identity (for example, becoming a patient rather than a surgeon), their work-life (for example, the need to give up work or work part-time), their career (being left behind as their colleagues progress), and the negative socio-emotional impacts on the female surgeon.

## Discussion

### Summary of Key Findings

Our study asked what gendered transitions female surgeons experience and the impacts of these transitions. We found that female surgeons’ multiple intersecting transitions were identified at different stages of training, challenging the notion of transitions as simple and linear. We adopted MMT theory to develop a holistic understanding of the complex multiple and multidimensional nature of transitions. Female gendered work, cultural and health transitions illustrated the sacrifices female surgeons made, with decisions severely impacting female surgeons’ careers as they tried to juggle family life, work-life and achievement of competencies. One transition often triggered other transitions, leading to a spiral of challenges for female surgeons.

### Comparison With Existing Literature

Our findings were reasonably consistent with existing literature, indicating transitions across the continuum of surgical training (4, 7–11, 16, 19), and with impacts on the individual and significant others (1). While some studies have previously explored the use of MMT theory as an analytical lens (5, 15, 16), our study was the first to use MMT theory to explore the multiplicity and multidimensional nature of female gendered transitions in surgical education.

Our study findings reported the socio-emotional impacts of leadership transitions on female surgeons when taking up senior roles as consultants. They showed a range of challenges for female surgeons at the consultant level, leading to several resigning very close to the end of their training. This is aligned with other literature, showing that unpreparedness for consultant positions and new clinical and non-clinical responsibilities (e.g., management, financial issues, and supervision) can lead to stress and burnout in doctors of all genders (27, 49, 50). While our study shows the impacts of transitions on females and their interpersonal relationships, other studies have shown unpreparedness for medical and generic competencies required for senior roles.

To our knowledge, our study is unique in that it is the first to explore the lived experiences of females who have left surgery. These participants reported the need for strong support networks. Several female surgeons reported how their transitions out of surgery interacted with their transitions to new medical careers. The need for mentorship for surgical trainees in decision-making around careers is consistent with the literature, which has reported that lack of social support influences women’s decisions to quit (21). However, we cannot make further comparisons with the literature because of limited research exploring gendered transitions in ex-surgeons.

Interestingly, numerous IMGs talked about the cultural transitions they experienced moving to work in Ireland and Scotland, necessitating integrating into new cultures. Previous research studies have described the need for cultural flexibility as a valuable attribute for adaptability to workplace changes in western cultures (51). Many doctors struggle with transitions to new workplaces, but IMGs have to make two major *shifts* in terms of (1) professional socialisation and (2) acculturation. IMGs have to learn the values, norms, and beliefs of the new society they are moving into and those of the medical profession as defined in the *new* country (52). Our study is novel in that it explored these issues for female IMGs through a gendered surgical lens.

Health transitions also triggered multiple and multidimensional life transitions, affecting surgical trainees’ self-confidence. These findings are consistent with Jindal-Snape’s work showing that some life transitions are not only triggered by health transitions but by a combination of other environmental factors (12). Our findings on female surgeons’ health transitions uniquely identified impacts on career progression and self-confidence. Importantly, our research shows that multiple transitions are individual, social and contextual processes, and further research needs to consider more the impacts of health transitions on female surgeons’ family members and colleagues, leading to burnout, compassion fatigue and secondary traumatic stress disorder (53, 54).

### Methodological Strengths and Limitations

We believe our study is the first to explore female gendered transitions and their impacts on diverse female surgeons from several surgical specialities, and drawing on MMT theory. A methodological strength of our study is that it explored conceptualisations in two healthcare systems, noting that we did not find any differences in female surgeons from these two countries. We employed a team-based approach to facilitate rigorous data analysis and interpretation of data. While there was diversity in the number of specialities represented, the sample size from each speciality was small, so it was impossible for us to explore any patterns by specialty. In addition, the sample sizes for the different subgroups (for example, female surgeons of colour, female ex-surgeons, etc.) were small, also prohibiting us from exploring patterns in our data.

### Study Implications

This study has both educational and research implications. Our key findings highlight female gendered multiple and multidimensional interacting transitions, as well as a multiplicity of impacts on self and others. Based on these findings, there is a need for educators, leaders and policy makers to understand the multiplicity of transitions and their impacts to firstly raise awareness. To minimise negative impacts, there is a need to develop and evaluate surgical transition interventions for surgical trainees and leaders (for example, by addressing female gendered transitions to senior roles). Policymakers need to develop services to meet female surgeons’ unique needs (for example, family-friendly structures and intercultural needs) as they transition through their surgical careers (see detailed recommendations aligned with the themes in the implications framework in Table 3). Regarding research implications, while our data collection explored participants’ surgical education journeys from a biographical narrative perspective, it did so from an interview at one snapshot in time. Therefore, further longitudinal investigation into the gendered transitions of female surgeons would be valuable to better understand in-the-moment changes across the surgical education journey to inform gender-related policies and practices. Indeed, longitudinal qualitative research considers time as fluid and accommodates changes in lived experiences through time (55). Finally, although our study identified female surgeons who transitioned out of surgery, female surgeons of colour, and female IMGs who transitioned to Ireland and Scotland for work, we had small numbers, so further research would benefit from a more thorough investigation of these under-represented groups.

**TABLE 3:** Recommendations for educators, leaders and policymakers based on themes.

Educators should:	Leaders should:	Policymakers should:
Provide educational interventions for female surgeons returning to surgery after maternity leave to re-build their surgical competencies	Offer support/mentorship to female surgeons returning to surgery after maternity leave, and recalibrate their expectations of returning surgeons’ performance	Ensure that females returning to surgery after maternity leave can access flexible working/training arrangements and childcare
Provide flexible options (e.g., online/blended learning) for educational interventions to ensure accessibility for female part-time surgical trainees	Respect part-time female surgeons’ working hours and ensure they are included fully in surgical teams despite working part-time	Ensure that part-time trainees are not discriminated against for training/working part-time and that their performance is judged based on achievement relative to opportunity
Provide bespoke leadership education interventions to female surgeons including gendered leadership issues	Be mindful of the impacts of becoming a female surgical consultant on trainees’ personal lives including significant others such as partners and children	Consider developing affirmative action recruitment policies for consultant surgeon posts to prioritise local female surgeons
Provide bespoke educational interventions to female surgeons changing training pathways including identity issues	Offer support/mentorship to female surgeons leaving surgery	Consider developing exit interview policies for women leaving surgery to better understand and improve surgical cultures for female trainees
Provide bespoke educational interventions to female surgeons changing countries, especially where cultural diversity exists between home-host countries	Respect female surgeons from other countries with different cultures and ensure they are included fully in surgical teams	Ensure that internationally qualified female surgical trainees are not discriminated against based on their cultural backgrounds, and consider developing affirmative action recruitment policies for consultant surgeon posts to prioritise cultural diversity
Provide educational interventions focusing on health and wellbeing for female surgeons experiencing physical and/or mental health problems or disability	Respect female surgeons experiencing ill health and ensure they are included fully in surgical teams	Ensure that female surgeons with ill health are not discriminated against, and can access flexible working/training arrangements if required, as well as adequate access to personal leave

## Data Availability Statement

The raw data supporting the conclusions of this article cannot be made available by the authors, because they do not have ethical approval to share these data. Further inquiries can be directed to the corresponding author.

## Ethics Statement

The studies involving human participants were reviewed and approved by the East of Scotland Research Ethics Committee (16/ES/0082), Galway University Hospital (C.A. 1697), The Adelaide and Meath Hospital, Dublin [2017-02 CA (16)], St. James Hospital, Dublin (Expedited approval), Sligo University Hospital (Expedited approval), University Hospital Limerick (064/17), and Beaumont Hospital (17/30). The patients/participants provided their written informed consent to participate in this study.

## Author Contributions

GO, SS, SC, and CR designed the study. GO obtained ethical approval, recruited the participants, collected the data, and wrote the manuscript. GO conducted this research as part of her Ph.D. at the Centre for Medical Education, University of Dundee, with SS, SC, and CR as her supervisors. All authors were involved in data analysis, edited, and commented on various iterations of the manuscript.

## Conflict of Interest

The authors declare that the research was conducted in the absence of any commercial or financial relationships that could be construed as a potential conflict of interest.

## Publisher’s Note

All claims expressed in this article are solely those of the authors and do not necessarily represent those of their affiliated organizations, or those of the publisher, the editors and the reviewers. Any product that may be evaluated in this article, or claim that may be made by its manufacturer, is not guaranteed or endorsed by the publisher.
